# Transcatheter Valve Replacement for Mitral Stenosis: A State of the Art Review

**DOI:** 10.3390/jcm15062373

**Published:** 2026-03-20

**Authors:** Alessandro Comis, Claudio Sanfilippo, Sebastiano Immè, Claudia Ina Tamburino, Luigi Ferrarotto, Antonino Salvatore Rubino, Corrado Tamburino

**Affiliations:** 1 Cardiology Unit, Policlinico Centro Cuore Morgagni, 95030 Pedara, Italy; alex.comis92@gmail.com (A.C.); claudiosanfilippo13@gmail.com (C.S.); sebymme@hotmail.it (S.I.); claudiatamburino@live.it (C.I.T.); luigi.ferrarotto@gmail.com (L.F.); antonio.rubino@hotmail.com (A.S.R.); 2 Faculty of Medicine and Surgery, Kore University of Enna, 94100 Enna, Italy

**Keywords:** mitral stenosis, mitral anulus calcification, TMVR

## Abstract

Degenerative mitral stenosis (MS) secondary to extensive mitral annular calcification (MAC) represents a growing clinical challenge in an aging population. These patients are often elderly, frail, and harbor a significant burden of comorbidities, rendering conventional mitral valve surgery prohibitively high-risk. While transcatheter mitral valve replacement (TMVR) has emerged as a potential alternative, the current evidence is only derived from single-arm observational registries. Therefore, the transition toward randomized controlled trials to define optimal patient selection and long-term prosthetic durability is necessary. This review examines the current landscape of TMVR for degenerative MS, focusing on the role of multimodal pre-procedural planning, procedural technique, and prevention of the principal complications. The integration of echocardiography and multi-slice computed tomography (MSCT) is essential for evaluating anatomical feasibility, particularly in predicting neo left ventricle outflow tract (neo-LVOT) obstruction, the primary determinant of procedural mortality. However, it is limited due to the absence of standardized protocol. We are showing the outcomes of off-label balloon-expandable aortic prostheses and dedicated TMVR system, which are the only two devices which data in patients with MS are available. Despite high technical success rates in specialized centers, complications, including paravalvular leak, valve thrombosis, and device migration, remain more prevalent than in aortic interventions. We present some tips and tricks to prevent and manage adverse events. TMVR represents a transformative frontier for inoperable patients with severe MAC. However, its routine clinical adoption requires further refinement of dedicated technologies and standardized imaging protocols to improve safety and bridge the gap between palliative medical therapy and definitive intervention.

## 1. Introduction

**Figure 1:**
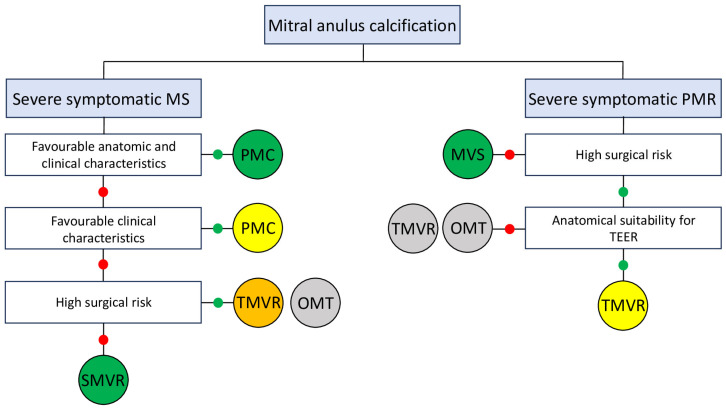
Mitral anulus calcification management. MS: Mitral stenosis; MVS: mitral valve surgery; OMT: optimal medical therapy; PMC: percutaneous mitral commissurotomy; PMR: Primary mitral regurgitation; SMVR: surgical mitral valve replacement; TMVR: Transcatheter mitral valve replacement; red dots mean no and green dots mean yes.

Transcatheter mitral valve replacement (TMVR) has emerged as a novel therapeutic option for patients with symptomatic mitral valve stenosis who are deemed to be at prohibitive risk for conventional surgery and possess an unfavorable anatomy for percutaneous commissurotomy. As the population ages, the incidence of degenerative mitral stenosis increases significantly. Patients presenting with mitral annular calcification (MAC) are typically elderly and carry a substantial burden of comorbidities, including disease of other valves. Moreover, MAC serves as an indicator of cardiovascular disease severity and is associated with an increased risk of atrial fibrilation (AF), stroke, and death [1,2,3]. Fortunately, in many cases, MAC does not result in significant valvular dysfunction [4]; however, in certain patients, calcific extension into the mitral valve leaflets or the subvalvular apparatus leads to clinically severe mitral stenosis, sometimes associated with combined regurgitation [5]. Current treatment options, including both transcatheter and surgical approaches, remain high-risk procedures, and evidence from randomized controlled trials is currently lacking. Degenerative mitral stenosis is generally not amenable to percutaneous mitral commissurotomy (PMC) because commissural fusion is absent, yet traditional cardiac surgery carries a high risk of mortality due to advanced age and comorbidities. In symptomatic, high-risk patients with suitable anatomy, the transcatheter implantation of a transcatheter aortic valve replacement (TAVR) prosthesis in the mitral position is feasible but is associated with frequent complications, including left ventricle outflow tract (LVOT) obstruction, valve embolization, stroke, and hemolysis due to paravalvular leak. Despite these interventions, mortality remains high. Consequently, the use of dedicated TMVR devices is encouraged, as they appear to offer a superior safety profile [6,7]. Reflecting the current literature, the latest European Society of Cardiology (ESC) guidelines for the management of valvular heart disease state that TMVR may be considered in symptomatic patients with extensive MAC and severe mitral valve dysfunction at experienced Heart Valve Centers with expertise in complex mitral surgery and transcatheter interventions [8]. Despite the availability of several multicenter registries, TMVR is not recommended for the treatment of mitral regurgitation in the new ESC guidelines, even in the presence of severe MAC. Therefore, in this patient population, the procedure remains off-label and should be reserved for patients with severe symptoms despite optimal medical therapy who are not candidates for Transcatheter edge to edge repair (TEER) or surgical intervention [8,9] (Figure 1).

In the present review, we aimed to outline the current challenges of the TMVR field, highlight the main considerations for TMVR planning, optimizing device selection and improving clinical outcomes in this high-risk patient population. Ultimately, we provide an overview of the current TMVR systems under clinical evaluation.

## 2. Pre-Procedural Planning

Pre-procedural assessment is a cornerstone to TMVR, integrating feasibility analysis with risk stratification for major complications, including device embolization and left ventricular outflow tract (LVOT) obstruction. The integration of imaging into procedural decision-making plays a key role to patients-tailored procedure. Advanced imaging guides device selection and sizing and informs critical procedural decisions, such as the optimal site of interatrial septal puncture and implantation height. This process is inherently multimodal, relying on complementary information from transthoracic and transesophageal echocardiography (TTE and TEE) and multi-slice computed tomography (MSCT) or cardiac magnetic resonance (CMR).

### 2.1. Echocardiography Assessment

**Figure 2:**
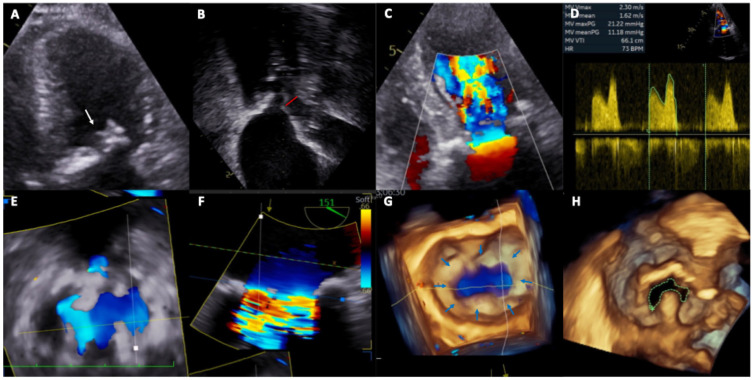
Pre-procedural echocardiography assessment. (**A**) Transthoracic echocardiography showed mitral leaflets and annular calcification (white arrow). (**B**) Transthoracic echocardiography left ventricle outflow tract assessment (red line). (**C**) Transthoracic echocardiography color aliasing showed mitral stenosis. (**D**) Transthoracic echocardiography Pulse wave Doppler showed severe mitral stenosis. (**E**) Transesophageal echocardiography showed severe anulus mitral calcification. (**F**) Transesophageal echocardiography color aliasing showed mitral stenosis. (**G**) 3D Transesophageal echocardiography showed circumferential mitral annular calcification (blue arrows). (**H**) 3D Transesophageal echocardiography showed reduced planimetric mitral valve area.

In TMVR in MAC, TTE is essential for the diagnosis of severe mitral stenosis, etiologic definition, and morpho functional valve assessment [10]. TEE using 3D imaging and color Doppler confirms the diagnosis and delineates critical pre-procedural landmarks, including atrial transseptal puncture height, interventricular septal hypertrophy, and left ventricular outflow tract gradient, while defining the quality of intraprocedural imaging available for transcatheter valve implantation guidance. A key disadvantage of echocardiography is its limited ability to distinguish calcification from fibrosis: although MAC typically appears as bright echo density with distal acoustic shadowing, leaflet fibrosis may mimic calcification, resulting in overestimation of MAC severity compared with cardiac computed tomography [11,12,13]. Furthermore, mitral valve dimensions and their relationships with adjacent structures are highly dependent on intravascular volume and hemodynamic status, imaging should be performed under conditions of normovolemia and hemodynamic stability (Figure 2).

### 2.2. Computer Tomography Assessment

**Figure 3:**
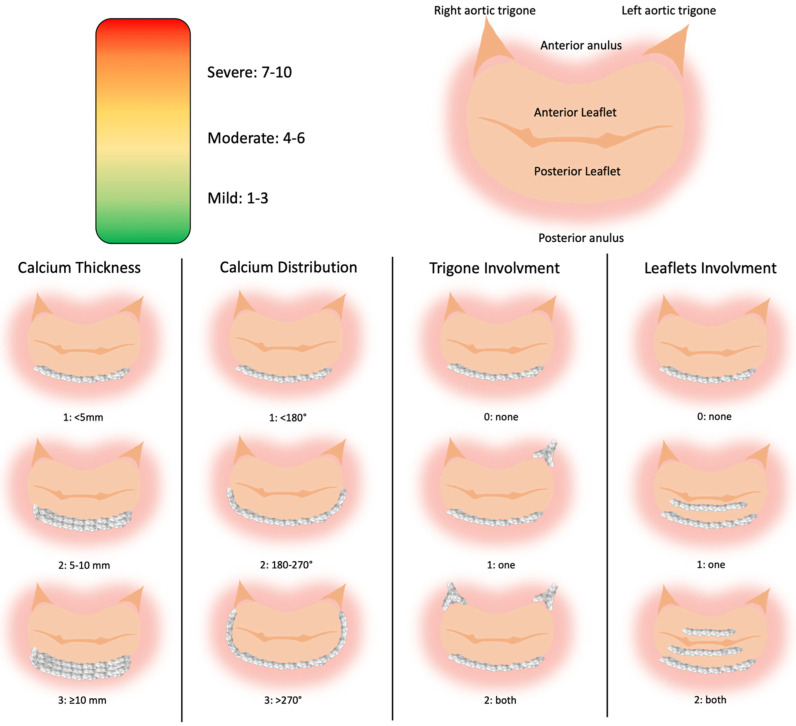
Mitral Anulus Calcification Score System. Elements used of the MAC severity score by Guerrero et al. [7]. Annular calcium thickness (<5 mm = 1 point; 5–10 mm = 2 points; ≥10 mm = 3 points); calcium distribution in annular circumference (<180° = 1 point; 180–270° = 2 points; >270° = 3 points), trigone calcification (none = 0 points; one = 1 point; both = 2 point), and mitral leaflet calcification (none = 0 point; one = 1 point; both = 2 point). A severity grade is assigned on the basis of total points accumulated as follows: mild MAC, 3 points or less; moderate MAC, 4 to 6 points; and severe MAC, ≥7 points.

MSCT plays a central role. Imaging should be acquired in both systolic and diastolic phases in normovolemia, with measurements performed in both phases to reduce bias. In systole, short-axis views allow for the assessment of mitral leaflet morphology [14]. In diastole, CT defines the spatial distribution of MAC, the mitral valve eccentricity, and adjacent structures; three-dimensional volume-rendering can give more information among calcium distribution [15]. MAC severity is determined by average calcium thickness (mm), circumferential annular involvement, calcification of one or both fibrous trigones, and leaflet calcification [16], and this scoring system also predicts the risk of embolization of balloon-expandable prostheses (Figure 3). Alternative approaches, including Agatston-based calcium scoring as used for TAVR, have been described but lack outcome correlation [17,18]. Furthermore, the extent of spicule calcifications in structures adjacent to the MAC such as the ventricle must also be assessed [19]. Diastolic-phase imaging is also used for annular sizing; however, to the nonplanar, saddle-shaped geometry of the mitral valve, accurate sizing requires 3D CT reconstruction [14].

**Figure 4:**
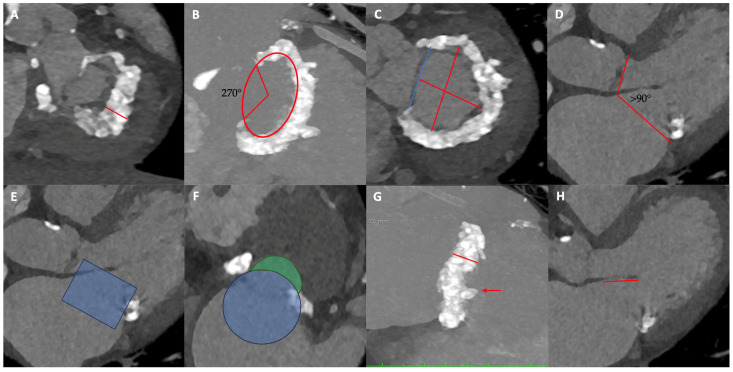
Pre-procedural computer tomography assessment. (**A**) Mitral leaflets and mitral anulus calcification thickness (red line). (**B**) Mitral annular calcification (MAC) extension (ranging from 0° to 360°). (**C**) Mitral valve plane demonstrating trigone to trigone distance (blue line), intercommissural distance (longer red line), and septo-lateral distance (shorter red line) which permit to measure the anulus eccentricity. (**D**) Aorto-mitral angle. (**E**) Neo-LVOT assessment. Virtual implantation of the transcatheter mitral valve (blue structure). (**F**) Minimal neo-LVOT area. Short axis demonstrating the virtually implanted device (blue circle) and the neo-LVOT assessment (green area). (**G**) Mitral anulus thickness (red line) and calcification spicula extended to left ventricle (red arrow). (**H**) Anterior mitral leaflet length.

Beyond evaluation of the mitral valve itself, careful assessment of its relationship with adjacent anatomical structures is required. In particular, it is essential to define the neo-left ventricular outflow tract (neo-LVOT) created by THV deployment and the displacement of the native anterior mitral leaflet towards the interventricular septum [20]. To predict the area of the neo-LVOT and estimate the risk of obstruction, a cylindrical polygon with the same dimensions of the selected prosthesis must be placed inside the mitral annulus in systolic scans. At this point, the area created between the lower frame of the polygon and the interventricular septum must be estimated (Figure 4E,F). A neo-LVOT area of ≤1.7 cm^2^ predicted LVOT obstruction with a sensitivity and specificity of 96.2% and 92.3%, respectively [21]. The principal predictors of neo-LVOT obstruction include aortomitral angulation close to 90°, severe septal hypertrophy (>15 mm), anterior mitral leaflet length, and THV implantation height, all of which should be carefully considered [22,23,24,25]. Depending on the estimated risk of neo-LVOT obstruction, alternative strategies may be pursued, including a more atrial device placement—at the expense of an increased risk of embolization and paravalvular leak—intentional laceration of the anterior mitral leaflet to create a flow window and enlarge the neo-LVOT [26], or septal myectomy to reduce hypertrophy [27]. Notably, some degree of valve oversizing is always advisable, reducing the risk of embolization and PVL. This fact determines flaring of the ventricular end of the prosthesis, which should be considered when defining the neo-LVOT area [28,29] (Figure 4). Fortunately, there are several tools that simplify CT implant simulations by reducing processing times and evaluation errors [23,30]. Finally, assessing the dimensions of the left heart chambers is crucial to determine their suitability for accommodating the mitral bioprostheses, the atrial transseptal puncture site, or the thickness of the cardiac apex when a transapical approach is planned [31]. For devices requiring apical anchoring, it is important to measure the distance between the MAC and the apex, as well as the myocardial thickness at the chordal insertion site [32].

Several disadvantages may influence the feasibility and the quality of the exam. First, the administration of iodinated contrast media is contraindicated in patients with advanced chronic kidney disease due to the risk of contrast-induced nephropathy. Second, mitral valve disease, specifically mitral stenosis, is frequently associated with atrial fibrillation; a rapid, irregular ventricular response can impair image acquisition quality by interfering with the synchronization between the ECG-gated system and the patient’s cardiac rhythm. Finally, while MSCT provides detailed morphological assessment, it lacks the capacity to provide comprehensive functional and hemodynamic data regarding the mitral valve. CMR is an alternative evaluation method to MSCT. This method may provide both morphological and functional information but has a poor ability to detect calcification [33].

## 3. Devices Under Clinical Evaluation

Percutaneous therapy for MS spans two fundamentally different device families: valve-sparing commissurotomy systems (best suited to pliable, commissural-fusion phenotypes) and valve-replacement strategies for degenerative/calcific MS. The characteristics, indications, and techniques for the use of valve-sparing commissurotomy systems are beyond the scope of this review. From a practical standpoint, currently used transcatheter mitral replacement technologies can be grouped into two categories: (i) dedicated TMVR platforms specifically engineered for the native mitral apparatus, and (ii) non-dedicated transcatheter heart valves originally developed for the aortic position that continue to be used off-label in selected mitral settings. Accordingly, the following section first summarizes key design features of contemporary dedicated TMVR systems and then briefly highlights commonly used non-dedicated balloon-expandable valves that remain part of current practice (Table 1).

### 3.1. Non-Dedicated TMVR Systems

When calcific MS is severe and anatomy is prohibitive for durable valvotomy, the dominant percutaneous replacement strategy has been valve in MAC (ViMAC) using balloon-expandable aortic THVs, leveraging the rigid calcific “landing zone” to stabilize the prosthesis. The early multicenter global registry of TMVR in severe MAC (predominantly using the SAPIEN XT/3 family, Edwards Lifesciences, Irvine, CA, USA) demonstrated feasibility but underscored the high-risk nature of this population, with substantial early mortality despite symptomatic improvement among survivors [34]. One-year follow-up from the same registry confirmed the trade-off profile: high 30-day and 1-year mortality overall, yet sustained hemodynamic performance (lower mean gradients, larger effective valve area) and functional improvement in those who survived beyond the early hazard period [35]. Real-world U.S. registry data further contextualized ViMAC outcomes relative to other transcatheter mitral indications, consistently showing that ViMAC carries higher adverse-event rates than valve-in-valve procedures—reinforcing the centrality of careful anatomic planning (including LVOT risk assessment) and meticulous implantation technique in this setting [37]. Alongside these “legacy” balloon-expandable platforms, newer balloon-expandable THVs such as Myval (Meril, Vapi, India) have entered the ViMAC space through early case-based experience, mainly supported by an expanded size matrix and contemporary sealing concepts; however, robust ViMAC-specific outcome datasets remain limited [41]. Because LVOT obstruction is a principal determinant of failure in ViMAC, the “device ecosystem” for calcific MS replacement includes not only the valve but also adjunctive tools designed to modify the outflow tract interaction. The earliest descriptions of LAMPOON established a transcatheter method to lacerate the anterior leaflet and mitigate LVOT obstruction risk during TMVR [42], and subsequent prospective and longer-term follow-up experience supports its role as a reproducible strategy in carefully selected anatomies where leaflet displacement is the dominant LVOT mechanism [26].

### 3.2. Dedicated TMVR Systems

A key limitation of non-dedicated ViMAC is that aortic THVs were not engineered for the mitral apparatus; therefore, dedicated TMVR systems aim to improve anchoring, sealing, and coaxial deployment while reducing reliance on circumferential calcific “purchase.” The Tendyne system (Abbott, Chicago, IN, USA) represents the most mature dedicated platform in severe MAC cohorts: it is a self-expanding, D-shaped mitral prosthesis stabilized by an apical tether and pad, enabling controlled seating and potentially less dependence on annular radial force. Early dedicated-MAC experience demonstrated feasibility of a purpose-built mitral prosthesis in this challenging disease spectrum [43], later expanded by broader early clinical experience showing meaningful symptom relief and stable valve function, albeit with the expected competing risks of mortality and heart-failure hospitalization in elderly, comorbid population [44]. More recently, the SUMMIT-MAC cohort provided the first prospective trial-level dataset supporting TMVR with Tendyne in severe MAC with mitral regurgitation or stenosis, demonstrating high technical success and clinically relevant improvements in outcomes and quality of life at 12 months [9]. The Intrepid TMVR system (Medtronic, Dublin, Ireland) (dual-stent, self-expanding architecture) has also reached calcific phenotypes through innovative “hybrid” enabling strategies. A pivotal illustration is the first-in-human report of IVL-assisted transseptal TMVR with Intrepid in a severely calcified mitral valve presenting with severe stenosis and regurgitation, highlighting how calcium modification can be used not only to facilitate valvotomy but also to permit complete prosthesis expansion and safer anchoring in extreme calcium burdens [45,46]. These experiences underscore an emerging design principle for calcific MS: the effective therapy is a “platform”, where valve choice, calcium-modification tools, and LVOT-mitigation techniques are co-optimized for each anatomy. Finally, fully transseptal dedicated TMVR platforms such as EVOQUE (Edwards Lifesciences, Irvine, CA, USA) (including its Eos iteration) have reported early feasibility and 1-year outcomes primarily in mitral regurgitation cohorts, reflecting a rapid field-wide move toward transseptal-only workflows [47,48]. However, peer-reviewed clinical outcome series specifically focused on MAC-driven stenosis/ViMAC using EVOQUE/Eos remain limited, and extending these platforms to calcific MS phenotypes should currently be regarded as investigational until dedicated datasets become available. Finally, the preliminary safety and efficacy of the Cephea mitral valve system (Abbott, Chicago, IN, USA) for the treatment of symptomatic patients with mitral valve disease, including patients with severe mitral stenosis, is being investigated in a prospective trial (NCT05061004). Other dedicated devices are also evaluated to management mitral regurgitation without stenosis [49].

## 4. Procedural Technique for Transcatheter Mitral Valve Replacement

The first in-human TMVR in MAC was performed in 2013 through open-surgery transapical approaches, following the first mitral valve in valve for degenerate bioprothesis surgical valve and the first valve in ring, performed in 2009 and 2011 respectively [50,51,52]. Then, with the development of technology and a delivery system, an alternative and less-invasive access site was found in the transfemoral approach, which permit it to cross the MAC through a transseptal puncture. The transapical method provides easy access to the mitral valve with great coaxially to deploy the prosthesis. However, it is more invasive and will have a negative effect at the left ventricle myocardium, with a negative effect on cardiac output [53]. While technical success has been reported with the transapical route in TMVR, it has been associated with a slight increase in procedural mortality compared to the transfemoral approach [54]; however, the transeptal access is now the preferred approach when the THV can be implanted in this modality. After obtaining large-bore femoral venous access, preferably under ultra-sound guidance [55,56], a transseptal puncture is commonly performed using a transeptal needle (Brockenbrough needle, Medtronic) in the infero-posterior fossa ovalis to optimize device delivery and reduce complications [57] under fluoroscopic and TEE or ICE guidance [58,59,60]. A dedicated transeptal sheath is then advanced into the left atrium, and the mitral valve is crossed using a coronary catheter, such as a Judkins Right or Amplatz Left, or both telescoped to enhance guidewire support, typically a hydrophilic wire [61]. Once the left ventricle is reached, the hydrophilic wire is exchanged with extra-stiff shaped wire, such as a Safari or Confida wire. In cases where an unfavorable angle exists between the inferior vena cava, the interatrial septum, and the mitral valve, a buddy-wire technique may be employed by positioning a second wire in the left ventricle to facilitate delivery system advancement; this secondary wire is removed prior to valve implantation [62]. Subsequently, balloon atrial septostomy is performed with a 12 mm or 14 mm balloon over a pre-shaped stiff wire positioned at the left ventricular apex. Guided by angles predicted on pre-procedural computed tomography and confirmed with live transesophageal echocardiography (TEE) and fluoroscopy, the transcatheter heart valve is advanced across the septostomy into the mitral annulus, ensuring optimal coaxial alignment. To assess the trackability of the delivery sheath along the anticipated trajectory a 12/14 mm balloon could be advanced across both the interatrial septum and the mitral valve [62]. The valve is then carefully deployed under rapid ventricular pacing to achieve an 80/20 position (80% ventricular). In select cases, pre-procedural interventions such as balloon valvuloplasty or intravascular lithotripsy have been utilized to modify a heavily calcified or rigid annulus, thereby facilitating smoother valve delivery, and reducing the risk of malposition [46]. Post-dilation may be required if paravalvular regurgitation is observed. Left ventricular outflow tract and mitral hemodynamics are then assessed with live TEE. Finally, the delivery system is retrieved, and femoral venous access is closed either manually, with single or double suture-based closure devices, or with surgical stitches.

## 5. Complications of Transcatheter Mitral Valve Replacement in Mitral Annular Calcification

### 5.1. Paravalvular Leak

Paravalvular leak (PVL) is primarily attributable to severe annular calcification and to the intrinsically elliptical geometry of the mitral annulus. Its incidence after valve implantation in the setting of mitral annular calcification (MAC) ranges from 5.7% to 15.7% [35,37]. Careful assessment and quantification of PVL are required both intra-procedurally and during follow-up. The diagnosis of PVL mandates a systematic transesophageal echocardiographic (TEE) evaluation, as transthoracic echocardiography may be inconclusive. The presence of PVL often necessitates post-dilatation after valve deployment. In addition to precipitating pulmonary congestion, paravalvular leaks, particularly those of mild severity, may lead to hemolysis, with an incidence after ViMAC reported to be as high as 17% at 1-year follow-up [4]. Residual significant PVL, especially when associated with symptoms or the need for blood transfusions, may require transcatheter PVL closure [63]. When neither surgery nor transcatheter intervention is feasible, medical therapy aims to mitigate symptoms and manage anemia [64]. To reduce the risk of PVL, a meticulous pre-procedural planning and exact programmed height implantation during the procedure should be conducted.

### 5.2. Thrombus Formation

Valve thrombosis is a multifactorial process driven by turbulent flow, low cardiac output with the resultant in reduction in leaflet motion, all of which promote platelet activation and thrombus formation [6,65]. Accordingly, the 2025 ESC Guidelines for Valvular Heart Disease recommend anticoagulation with vitamin K antagonists (VKAs) for at least 3 months [8,66]. Thrombus formation may lead to heart failure, owing to impaired leaflet mobility, as well as thromboembolic complications. Serial transthoracic echocardiography (TTE) and scheduled follow-up assessments enable early detection and management of valve thrombosis. TTE may raise suspicion of thrombosis through the identification of limited leaflet mobility, cusp thickening, a ≥50% increase in mean transvalvular gradient, or, less commonly, direct visualization of thrombus. In most cases, the diagnosis is established by transesophageal echocardiography (TEE) or multidetector computed tomography (MDCT) [67,68]. Management depends on thrombus size and hemodynamic impact and relies on anticoagulation with VKAs, surgical removal, or percutaneous aspiration if surgery is not feasible [8,69,70,71,72]. The use of VKA, for at least the first three months after the index procedure, helps prevent thrombus formation, as recommended by the guidelines [8].

### 5.3. Device Migration and Embolization

The mitral valve is a dynamic and complex structure, and its morphology can influence the performance of an otherwise optimally implanted device. Suboptimal delivery, incomplete anchoring to the annulus, or incorrect device selection or sizing may result in acute or delayed malposition or migration. Careful pre-procedural evaluation is therefore essential to determine procedural feasibility and to ensure appropriate device selection. In addition, serial echocardiographic and computed tomographic imaging is required to assess the long-term efficacy of TMVR [73]. As for PVL a correct planification and device implantation may reduce the risk of embolization.

### 5.4. Atrioventricular Rupture

Atrioventricular rupture is a rare but catastrophic adverse event which may occur during a TMVR. Asymmetric MAC, small ventricular dimensions, valve oversizing, and aggressive dilatation are risk factors for atrioventricular injury [73].

### 5.5. Obstruction of the Left Ventricular Outflow Tract

Left ventricular outflow tract (LVOT) obstruction is caused by the atrioventricular positioning of the bioprosthesis, which displaces the anterior mitral leaflet toward the interventricular septum, resulting in a neo-LVOT that is smaller than the native outflow tract. In some cases, LVOT obstruction is dynamic, arising from a Venturi effect generated within the neo-LVOT that draws the anterior mitral leaflet toward the septum during systole, thereby producing dynamic obstruction. This complication leads to hemodynamic compromise and represents the most common cause of mortality in these patients [26,35]. In patients at high risk for LVOT obstruction, preventive strategies may be employed, including reduction in septal thickness (through alcohol septal ablation or percutaneous myectomy) [27,74] or intentional laceration of the anterior mitral leaflet (LAMPOON or BATMAN technique) [75,76,77].

## 6. Discussion

The convergence of an aging population and increased life expectancy has catalyzed a clinical shift in which degenerative mitral stenosis, secondary to extensive mitral annular calcification (MAC), is no longer a rarity but a growing epidemic among the elderly. These patients are typically characterized by significant frailty and a high burden of comorbidities that render traditional surgery prohibitive and percutaneous mitral commissurotomy unfeasible [78]. Historically, such patients were relegated to medical therapy, used only to manage symptoms and pulmonary congestion without modify natural history of the disease [79]. In response to this therapeutic vacuum, the scientific community has explored transcatheter alternatives, often drawing parallels with the success of aortic valve interventions. However, the off-label implantation of aortic prostheses in the mitral position is fraught with challenges due to anatomical differences between the two valves. While the aortic valve features a relatively simple, nearly circular annulus with low eccentricity, the mitral apparatus is a highly complex, elliptical structure. Its asymmetric and unevenly distributed calcifications impede the achievement of an adequate prosthetic seal, thereby increasing the risks of paravalvular leakage and device instability [80]. Furthermore, while the aortic valve is easily accessible via a transfemoral route, the mitral valve necessitates more complex transseptal or transapical approaches, significantly elevating periprocedural risk. Perhaps the most formidable technical barrier in transcatheter mitral valve replacement (TMVR) is the risk of left ventricular outflow tract (LVOT) obstruction. These limitations explain why TMVR has not experienced the same rapid expansion as transcatheter aortic valve replacement (TAVR) [81]. Consequently, recent European guidelines for the management of valvular heart disease provide a guarded recommendation for TMVR in symptomatic patients with severe MAC, reflecting the current scarcity of high-level evidence and randomized controlled trials. The transition from surgical ineligibility to procedural success is therefore predicated on an exhaustive, multimodal pre-procedural assessment [8]. The integration of transthoracic and transesophageal echocardiography with multi-slice computed tomography (MSCT) and, in selected cases, cardiac magnetic resonance (CMR) has become the cornerstone of contemporary TMVR. This imaging triad is indispensable for the precise quantification of the “Neo-LVOT” area and the critical determination of the most suitable device, whether an off-label balloon-expandable valve or a dedicated TMVI system. Ultimately, the technical success of TMVR is not merely a matter of device delivery but a sophisticated exercise in anatomical navigation and risk mitigation, necessitating specialized Heart Valve Centers with integrated multidisciplinary expertise.

## 7. Future Prospective

The evolution of TMVR in the coming years will focus on several hot topic. First of all, the future lies in overcoming the “off-label” use of aortic valve prostheses. Dedicated TMVR devices with lower profiles are needed to reduce left ventricular bulk and specific anchoring systems for the calcified annulus (MAC), minimizing the risk of migration and paravalvular leak (PVL). To date, only one dedicated device with transapical access has been evaluated for the treatment of calcific mitral stenosis. Several dedicated TMVR devices are available on the market and may be used in the future to treat MAC with stenosis in inoperable patients. Furthermore, alongside established leaflet-splitting techniques to prevent LVOT obstruction, the development of annular-remodeling technologies like intravascular lithotripsy will be pivotal. By enhancing prosthetic sealing within severely calcified frameworks, these approaches could expand TMVR eligibility to high-risk populations currently deemed anatomically ineligible. Third, improving outcomes will require the identification of diagnostic algorithms that allow the optimal timing of treatment in patients who are most likely to benefit from TMVR. Multimodality imaging will play a key role in patient and device selection. The integration of artificial intelligence and dedicated planning tools, similar to those already used in TAVR planning, may further facilitate this process, and increase its accessibility for clinicians. Finally, Currently, the available literature consists only of single-arm studies assessing the feasibility and safety of the procedure, without comparison with surgical intervention. Therefore, comparative studies and randomized clinical trial evaluating TMVR versus surgery in patients with MAC, as well as TMVR versus TEER in cases of mitral regurgitation without stenosis, are warranted. Direct comparisons of TMVR, conventional surgery (where possible), and optimal medical therapy will provide the basis for more robust guidelines and for defining the optimal timing of intervention before myocardial damage becomes irreversible.

## 8. Conclusions

In conclusion, TMVR is emerging as an essential therapeutic frontier for patients with degenerative mitral stenosis and severe MAC. Although significant anatomical challenges remain, refinement of multimodal pre-procedural planning and the advent of dedicated technologies promise to improve clinical outcomes. The transition from “rescue” procedures to standardized interventions, however, will require rigorous validation through randomized clinical trials and a continued commitment to innovation in prosthetic design.

## Figures and Tables

**Table 1:** Clinical studies of device used for mitral stenosis in mitral anulus calcification.

First Author, Year	PMID	Design	Number of Patients	Device	Access Site	Technical Success	Mortality
Guerrero, 2016 [34]	27388824	Multicenter retrospective registry	64	SAPIEN XT	Transatrial, transapical, transseptal	72%	30-day: 29.7%
Guerrero, 2018 [35]	29699609	Multicenter registry	116	SAPIEN XT	Transatrial, transapical, transseptal	-	30-day: 25%; 1-year: 53.7%
Yoon, 2019 [36]	30357365	Multicenter registry	58	SAPIEN XT, SAPIEN 3	Transseptal	62.1%	30-day: 34.5%; 1-year: 62.8%
Guerrero, 2020 [37]	32138529	National registry	100	SAPIEN 3	Transatrial, transapical, transseptal	74%	In-hospital 18%; 30-day 21.8%
Guerrero, 2021 [38]	33888229	Prospective multicenter	31	SAPIEN XT, SAPIEN 3	Transatrial, transapical, transseptal	74.2%	30-day 16.7%; 1-year 34.5%
Wild, 2022 [6]	35064722	Retrospective registry	108	Tendyne	transapical	94%	30-day: 12%
Guerrero, 2023 [7]	37758379	Prospective multicenter registry	91	SAPIEN XT, SAPIEN 3	transseptal, transapical, transatrial	-	5 years: 67.9%
Brener, 2024 [39]	36153166	Multicenter registry	126	SAPIEN XT, SAPIEN 3, SAPIEN 3 Ultra	Transatrial	94.4%	30-day: 12.7%; 1-year: 26.2%
Sorajja, 2026 [9]	41194751	Prospective clinical trial	103	Tendyne	transapical	94.2%	30-day: 6.8%.
[40]	NCT05061004	Prospective clinical trial	Estimates 50	Cephea	transseptal	-	-

## Data Availability

Data are available from the corresponding author on reasonable request.

## Abbreviations

TMVR	Transcatheter mitral valve replacement
MAC	Mitral anulus calcification
ViMAC	Valve in Mitral anulus calcification
